# Position dependent mismatch discrimination on DNA microarrays – experiments and model

**DOI:** 10.1186/1471-2105-9-509

**Published:** 2008-12-01

**Authors:** Thomas Naiser, Jona Kayser, Timo Mai, Wolfgang Michel, Albrecht Ott

**Affiliations:** 1 Experimentalphysik I, Universität Bayreuth, D-95440 Bayreuth, Germany; 2 Experimentalphysik, Universität des Saarlandes, D-66041 Saarbrücken, Germany

## Abstract

**Background:**

The propensity of oligonucleotide strands to form stable duplexes with complementary sequences is fundamental to a variety of biological and biotechnological processes as various as microRNA signalling, microarray hybridization and PCR. Yet our understanding of oligonucleotide hybridization, in particular in presence of surfaces, is rather limited. Here we use oligonucleotide microarrays made in-house by optically controlled DNA synthesis to produce probe sets comprising all possible single base mismatches and base bulges for each of 20 sequence motifs under study.

**Results:**

We observe that mismatch discrimination is mostly determined by the defect position (relative to the duplex ends) as well as by the sequence context. We investigate the thermodynamics of the oligonucleotide duplexes on the basis of double-ended molecular zipper. Theoretical predictions of defect positional influence as well as long range sequence influence agree well with the experimental results.

**Conclusion:**

Molecular zipping at thermodynamic equilibrium explains the binding affinity of mismatched DNA duplexes on microarrays well. The position dependent nearest neighbor model (PDNN) can be inferred from it. Quantitative understanding of microarray experiments from first principles is in reach.

## Background

The well-known double-helix structure of nucleic acids results from sequence-specific binding between complementary single strands. Sequential base pairing between A·T and C·G base pairs along the two complementary strands results in the formation of stable duplexes. This so called hybridization process is fundamental to many biological processes and biotechnologies. Microarrays consist of surface-tethered *probe *sequences, which act as specific scavengers for their respective complementary *target *sequence. The molecular recognition enables a highly parallel detection of nucleic acid sequences in complex target mixtures. Hybridization also occurs with single mismatched (MM) base pairs, however, these duplexes are significantly less stable than the corresponding perfect match (PM) [1,2]. The single base pair mismatch-discrimination capability of short (~20 nt) oligonucleotide probes provides an important diagnostic tool for the detection of point-mutations and single nucleotide polymorphisms (SNPs) [3]. DNA duplex stability arises from hydrogen bonding and base stacking interactions (the latter comprise van der Waals interactions, electrostatic and hydrophobic interactions between adjacent base pairs). According to the well-established nearest-neighbor model, thermodynamically a nucleic acid duplex can be considered the sum of these nearest-neighbor (NN) interactions [4-6]. The binding free energy of an oligonucleotide duplex can be predicted from the nearest-neighbor free energy parameters: The helix propagation parameters (one for each of the 10 possible base-pair doublets in case of a DNA/DNA duplex) account for the duplex sequence. Further parameters provide corrections for duplex initiation, A·T terminal pairs or a symmetry penalty in case of self-complementary sequences. The NN model adequately predicts oligonucleotide duplex melting temperatures *T*_*M *_in bulk solution [7]. Datasets of Watson-Crick NN parameters [8] provide the basis for nucleic acid structure and melting temperature prediction software like the DINAMelt web server [9] (UNAFold), the HYTHER server and others. The NN model can be extended beyond the Watson-Crick pairs to include single base MM defects [7,10].

In spite of good knowledge about nucleic acid hybridization in solution, the prediction of binding affinities on DNA microarrays remains empirical. Recent microarray studies [11-15] report, that the influence of even a point defect on hybridization signal intensity cannot be predicted easily. In particular the influence of defect position on the hybridization signal is stronger than the influence of MM-type [12,14,16].

Experiments show that the two-state nearest-neighbor (TSNN) approach [7], which has been very successful in predicting duplex stability in solution, does not appropriately describe MM binding affinities on DNA microarrays. The NN model does not account for the position of the individual NN pairs [7], except for the outermost ones. Based on microarray data, Zhang *et al*. [17] proposed a position dependent nearest-neighbor (PDNN) model. The model assumes that the duplex binding free energy can be expressed as a weighted sum of stacking energies with empirically derived positional weight parameters [17-21]. The purpose of this study is to investigate the influence of point defects on (surface bound) hybridization experimentally and theoretically. Previous studies investigate mismatch discrimination with samples of very different sequence motifs [11,12]. However, other effects such as secondary structure formation or competitive binding may reduce the visibility of the impact of the MM-defect on the binding affinity. To avoid such complications we performed experiments with fixed sequence motifs: We focus on small variations of the probe sequences. We perform hybridization studies with home-made microarrays comprising sets of very similar probe sequences. We use a single target sequence in each hybridization assay in order to avoid inter-target binding as well as target competition of different sequences for one and the same probe sequence. In order to avoid excluded volume interactions or secondary structure we limit the length of the target sequence to be of the order of the probes. These simplifications (described in detail in [14]) enable a detailed investigation of the influences of defect type, defect position, flanking base pairs and the sequence motif on the binding affinity. The extensive set of hybridization affinities obtained from our experiments enables us to perform a very complete analysis. We compare the experimental data to theoretical modeling based on a double-ended molecular zipper approach (the double-ended nucleic acid zipper has been previously described by [22-26]). We find that in order to reproduce the microarray hybridization signal in our model, the heterogeneity of binding affinities – mostly owing to *in situ *synthesis-related probe defects (e.g. probe polydispersity) – needs to be taken into account. More than that, synthesis defects arise as useful for parallel detection of many different sequences.

## Methods

### DNA Microarray Hybridization Experiments

**Figure 1:**
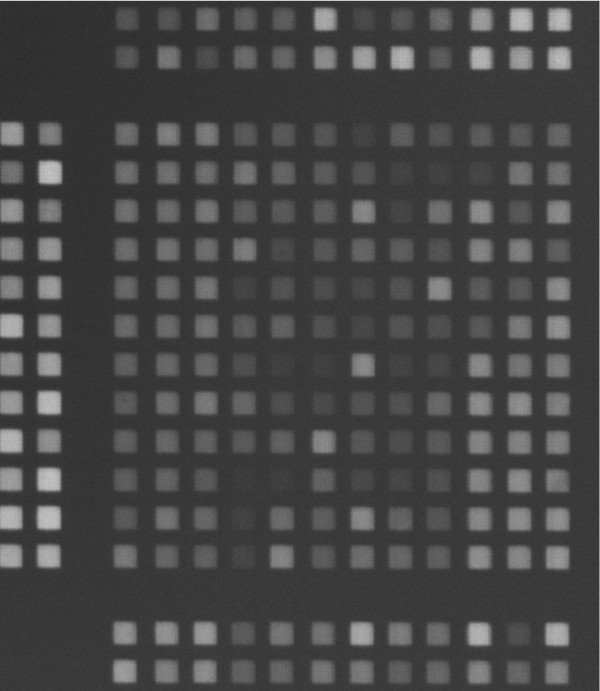
**Fluorescence micrograph (taken with an Olympus IX81 epi-fluorescence microscope and a Hamamatsu EM-CCD camera) of a microarray feature-block comprising variations of the 16 mer probe sequence motif 3'-TATTACTGGACCTGAC-5'**. Microarray hybridization was performed with the 5'-Cy3-labeled RNA oligonucleotide target 3'-AACUCGCUAUAAUGACCUGGACUG-5' (target concentration: 1 nM in 5 × SSPE, pH 7.4, 0.01% Tween-20, *T *= 30°C). Each 3 × 3 sub-array comprises (randomly arranged) one perfect matching probe, three single base mismatch probes, four insertion probes and one single base deletion probe. In Fig. 2A the hybridization signals (fluorescence intensities, averaged over the center of the microarray features) are plotted versus the defect position. The size of each microarray feature is 21 μm and the pitch of the array is 35 μm. The significantly brighter feature-block at left comprises variations of the 20 mer probe sequence motif 3'-TTGAGCGATATTACTGGACC-5'.

Hybridization assays are performed on high-density oligonucleotide microarrays (see Fig. 1). These microarrays (DNA Chips) are fabricated in-house [14] on the basis of light-directed solid-phase combinatorial chemistry [27,28]. A "maskless" photolithographic technique [13,29-31] based on a digital micromirror device type spatial light modulator (DMD™, Texas Instruments Inc.) enables tailor-made design of DNA microarrays (with up to 25000 different probe sequences) on a laboratory scale. Point defects – single base substitutions, insertions and deletions – are produced in the *in situ *synthesis process by variation of the nucleotide coupling scheme for the particular probe sequence.

Protocols for the preparation of dendrimer-functionalized microarray substrates (adapted from [32]) and for the light-directed synthesis (based on NPPOC-phosphoramidites [33]), as well as details on the hybridization assay and on fluorescence microscopy based microarray analysis (Fig. 1) are provided in Naiser *et al*. [14,15].

In each microarray hybridization assay a probe set of cognate probes with purposefully introduced point mutations – derived from a common probe sequence motif – is hybridized against a single target sequence, which perfectly matches the probe sequence motif. We systematically vary defect type and defect position to provide the complete "defect profile" of hybridization affinities with probe sets. We include not only all single base mismatches (MMs), but also, in order to investigate mismatch discrimination in a broader context of other sequence defects, we consider single base bulges (originating from insertions and deletions) as well as probes with multiple defects. Since the ca. 130 probes within each probe set differ only by single bases we are able to distinguish between defect-positional and sequence influence. In our experimental conditions hybridization equilibrium is reached after a few tens of minutes. Further details can be found in [14].

## Results

### Position Dependent Influence of Single Base MMs and Bulges on Probe-Target Binding Affinity

**Figure 2:**
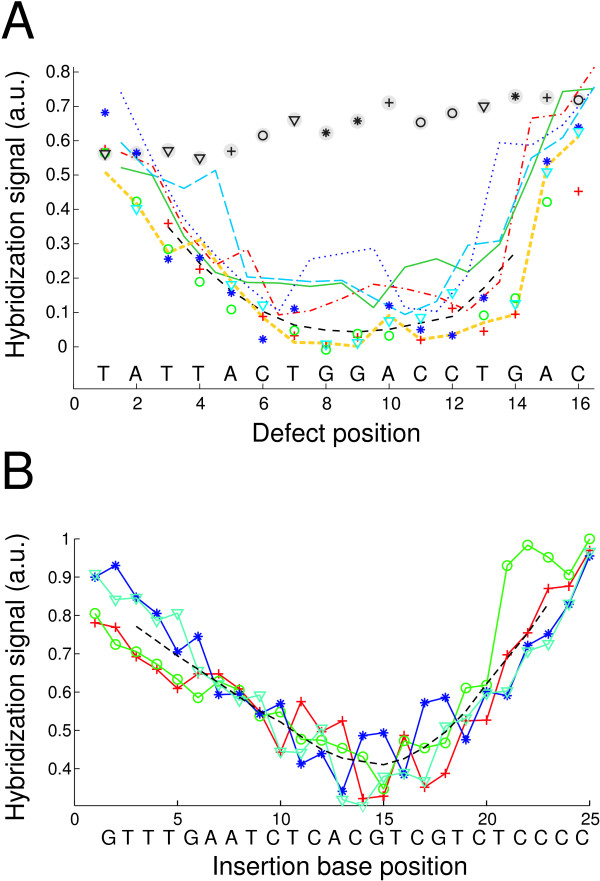
**The "defect profile" shows the position-dependent impact of single base mismatches, insertions and deletions on hybridization affinity**. Symbols: MM probes with substituent bases A (*red crosses*), C (*green circles*), G (*blue stars*), T (*light blue triangles*); moving average of all MM intensities (*black dashed curve*); single base insertion probes with insertion bases A (*red dash-dotted curve*), C (*green solid curve*), G (*blue dotted curve*), T (*light blue dashed curve*). Defect profiles of different probe sequence motifs. (*A*) Position dependent impact of various single base defects on the hybridization affinity for the probe sequence motif 3'-TATTACTGGACCTGAC-5' (hybridized with the complementary RNA target sequence). Hybridization signals of single base deletions (*orange dashed curve*) are comparable to that of MMs at the same position. PM probe signal replicates (*black symbols on gray ground*) serve as an indicator for spatial bias on the microarray. Deviations of MM hybridization signals from the mean profile are mostly MM-type specific. Increased hybridization signals of certain insertion probes (where the bulged surplus base is located next to identical bases – Group II bulges [14,53]) are due to positional degeneracy of the bulge defects. (*B*) Position dependent impact of various single base insertion defects on the hybridization affinity for the probe sequence motif 3'-GTTTGAATCTCACGTCGTCTCCCC-5' (hybridized with the complementary DNA target sequence). Insertions of A (*red crosses*), C (*green circles*), G (*blue stars*), T (*light blue triangles*), moving average of all insertion probe intensities (*black dashed curve*). Systematically increased hybridization signals of Group II bulges are discussed in Additional file 1, Fig. S2.

From the fluorescence micrograph (Fig. 1), we obtain the hybridization signals, which we plot as a function of defect position (Fig. 2). We note a strong influence of the defect position on probe-target binding affinity which is larger than the influence of the defect type. We find that bulge defects display a very similar position-dependent influence on hybridization signal intensity to mismatches. Furthermore we observe that the magnitude of mismatch discrimination (and bulge discrimination) at a particular defect position (i.e. the shape of the defect profile) depends on the duplex sequence.

As can directly be inferred from Fig. 2, defects in the middle of the probes are most destabilizing. In the center of a 16 mer duplex a single nucleotide MM typically reduces the hybridization signal to 0–40% of the corresponding PM duplex hybridization signal. Defect type and nearest-neighbor effects have less influence on the hybridization signal than defect position. Our experiments show a mostly monotonous decrease of hybridization signals over a range of typically 5–8 defect positions (for 16 mer probes and up to 14 positions for some 25 mer sequence motifs) from the duplex ends towards the center of the duplex. This is consistent with previous work [11,12].

**Figure 3:**
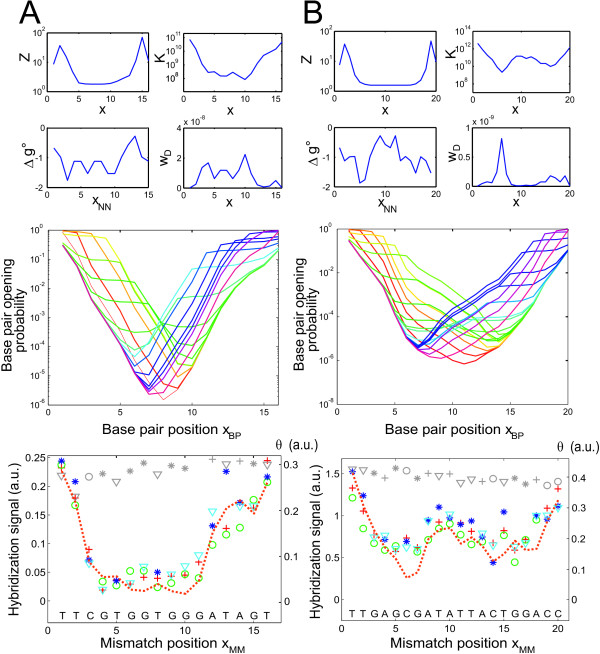
**Comparison of simulation results with the experimentally determined hybridization affinities for two probe sequence motifs (*A*) and (*B*)**. The four small sub-figures in the top section (from *top left *to *bottom right*) show the partition function *Z *and the duplex binding constant *K *as a function of defect position *x *(semi-logarithmic plots), the NN-free energies Δ*g*° of particular NN-pairs as a function of NN-pair position *x*_*NN*_, and the statistical weight for complete duplex dissociation w_*D *_as a function of defect position. Irregularities in *Z*(*x*) at the duplex ends are an artifact caused by the fact that only a single NN-pair is affected by a MM-base pair at the duplex end. The middle sub-figure shows the base pair opening probabilities (the fraction of strands in which the corresponding base pair at position *x*_*BP *_is unzipped) as a function of the defect position. The spectrum of differently colored curves encodes the different defect positions *x*_*MM *_(*red *– defect at left end; *purple *– defect at right duplex end). The bottom sub-figure compares the experimentally determined MM defect profile (mismatched base: A (*red cross*), C (*green circle*), G (*blue star*), T (*cyan triangle*); *gray symbols *correspond to PM probes) with the simulated MM defect profile *θ*(*x*) (*dashed orange line*). With Δ*g*_*def *_= 1 kcal/mol (at the simulation temperature of 325 K) and an error rate of 12 percent (per synthesis step) the calculated defect profile *θ*(*x*) matches well the experimental data.

In order to separate the defect positional influence (DPI) for a particular probe sequence motif from the defect type related influences we run a moving average filter on the defect profile. We observe that the DPI is not only a simple function of the distance between the defect and the duplex-ends, but it is also related to the nucleotide sequence (compare Fig. 2A and 2B and Fig. 3A and 3B).

We also perform hybridization experiments on oligonucleotide duplexes with two single base deletion defects at varying positions *x *and *y*. The results show that the binding affinity depends also on the relative position of the defects (for details see Additional file 1, Fig. S5 and [26]). The hybridization signal is largest if each defect is located close to an end. Lowest binding affinities are observed for defect configurations which divide the sequence into three roughly equally long subsequences. Closely spaced defects (with a distance of less than four nucleotides) systematically increase their impact with distance.

## Discussion

Single base mismatches and base bulges alike show a strong, trough-shaped position-dependent influence biased by the considered sequence motif. Experimental evidence for an influence of the sequence context (beyond the nearest neighbors) on the stability of single base pair MMs has been reported previously (hybridization of short 31 bp linear oligonucleotide duplexes in bulk solution) by Benight and coworkers [34], however such effects have not yet been systematically quantified. The commonly used two-state model of nucleic acid hybridization between the microarray probe *P *and the target strand *T *resulting in the formation of the duplex *D *is described by Eq. 1.

(1) $$P+T\overset{k_{nuc}}{\underset{k_{diss}}{⇌}}D$$

In thermodynamic equilibrium duplex nucleation (determined by the slow nucleation rate *k*_*nuc*_) is balanced by duplex dissociation with the dissociation rate *k*_*diss*_. The widely used two-state nearest-neighbor model (including mismatched NN-dimers as described by [10]) cannot provide an explanation for this positional influence, it does not account for the position of the individual nearest-neighbor dimers. We assume that the nucleation rates *k*_*nuc *_of very similar duplexes (differing by a single base pair, e.g. a PM duplex and a corresponding mismatched duplex) are virtually identical. Thus, the positional dependence observed experimentally can be expected to result from differences in *k*_*diss*_. In agreement with [25] we show that the positional influence originates from end-domain unzipping. Our experimental findings suggest a common mechanism for DPI, that is independent of the defect type. Further, the relatively long range of the DPI (Fig. 3A and 3B) suggests that molecular dynamics may well be a good candidate for an explanation. The symmetry of DPI (with respect to the duplex ends) and sequence-specific deviations from the symmetry indicate a zipping related mechanism. Thus, in order to account for partial denatured duplex states, we use a double-ended zipper model of the oligonucleotide duplex to determine mismatched oligonucleotide duplex stabilities as a function of defect position. We consider a situation in thermodynamic equilibrium.

### Double-ended Zipper Model

**Figure 4:**
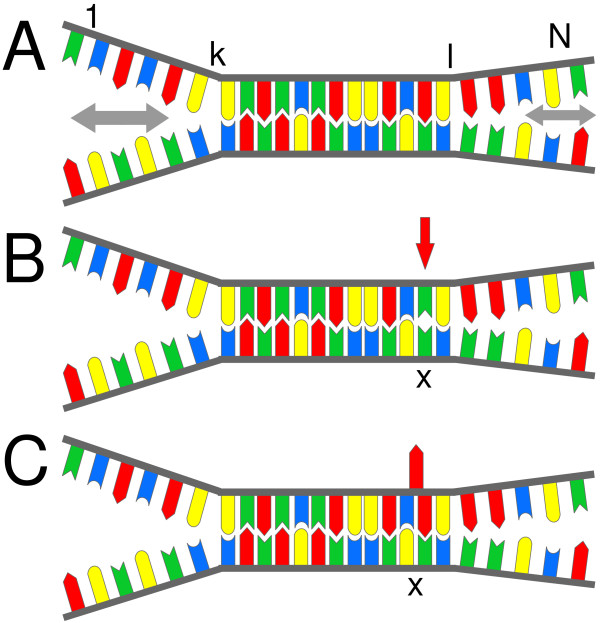
**Double-ended zipper model of the oligonucleotide duplex**. (*A*) Unzipping of the relatively short duplexes is initiated at the duplex ends only. The end-domain opening, which progresses back and forth (nucleotide by nucleotide) in a stepwise, zipper-like fashion, can be considered a biased random walk. The energy level of the partially denatured hybridization state *S*_*k*,*l *_(with respect to the completely hybridized ground state) is determined by summation over the NN free energies of the unzipped NN-pairs (from 1 to k and from l to N). (*B*) Single base MMs (non-Watson-Crick base pairing affect the stabilities of two adjacent NN-pairs at positions *x *and *x *+ 1. (*C*) Base insertions and deletions result in bulged duplexes with an unpaired base. The surplus base (depicted in a looped out conformation), similar as a MM defect, results in a reduced binding affinity.

We check if a double-ended zipper model [22-25] (Fig. 4), considering end-domain-denaturation only, is appropriate to describe the experimental observations. Internal denaturation, due to the large bubble initiation barrier (owing to stacking interactions towards both sides of a nucleotide) and due to the relatively short length of the duplexes, is expected to be negligible [22]. Using a partition function approach the impact of point defects is investigated at thermodynamic equilibrium. We perform this analytically, independently of a particular sequence, as well as numerically with sequence-dependence – using unified NN-parameters [8].

According to Craig *et al*. [35] a kinetic scheme describing helix growth and dissociation is given in Eq. 2.

(2) $$P+T\overset{k_{nuc}}{\underset{k_{-}}{⇌}}D_{1}\overset{k_{+}}{\underset{k_{-}}{⇌}}D_{2}\overset{k_{+}}{\underset{k_{-}}{⇌}}...\overset{k_{+}}{\underset{k_{-}}{⇌}}D_{n-1}\overset{k_{+}}{\underset{k_{-}}{⇌}}D_{n}$$

*k*_+ _and *k*_- _are the fast zipping and unzipping rates determined by the nearest-neighbor propagation parameters of the individual base-pair doublets. The time-evolution of the oligonucleotide zipper can be considered a biased random walk with a finite probability for complete dissociation (described by the duplex dissociation rate *k*_*diss*_). Since we consider thermodynamic equilibrium, we can use a partition function for fast numerics.

### Partition Function Approach (PFA) to Investigate Oligonucleotide Duplex Thermodynamics

We use a partition function approach [22-25] and investigate if the double-ended zipper model can reproduce our experimental results. On the basis of unified NN-Parameters [8] we calculate statistical weights of partially denatured duplex states. The effect of partial binding with respect to microarray data was discussed earlier in [24-26].

The partition function Z_*D *_of the duplex (Eq. 3) is the sum of the statistical weights w_*k*,*l *_of all partially hybridized duplex states S_*k*,*l *_(see Fig. 4).

(3) $$Z_{D}=\sum_{k=0}^{N-1}\sum_{l=k+1}^{N}w_{k,l}=\sum_{k=0}^{N-1}\sum_{l=k+1}^{N}e^{\Delta G_{k,l}^{\circ }/RT}$$

The statistical weight *w*_*k*,*l *_of the partially denatured state S_*k*,*l *_is calculated from the sum $\Delta G_{k,l}^{\circ }$ of NN free energies $\Delta g_{i}^{\circ }$ of the unzipped duplex sections (Eq. 4). $\Delta G_{k,l}^{\circ }$ can be considered as the free energy level of the partially denatured state.

(4) $$\begin{array}{l} \Delta G_{k,l}^{\circ }=\sum_{i=1}^{k}\Delta g_{i}^{\circ }+\sum_{i=l+1}^{N}\Delta g_{i}^{\circ }\\ \begin{array}{cc} \Delta G_{0,l}^{\circ }=\sum_{i=l+1}^{N}\Delta g_{i}^{\circ } & \Delta G_{k,N}^{\circ }=\sum_{i=1}^{k}\Delta g_{i}^{\circ } \end{array} \end{array}$$

NN free energies of Watson-Crick NN-pairs are deduced from unified NN parameters [7].

(5) $$\Delta g_{i}^{\circ }=\Delta h_{i}^{\circ }-T\cdot \Delta s_{i}^{\circ }$$

For the completely dissociated duplexes we estimate partitions functions of probes Z_*P *_and targets Z_*T *_as

(6) $$\begin{array}{cc} Z_{P}=Z_{T}=e^{\Delta G_{D}^{\circ }/(2RT)} & \Delta G_{D}^{\circ }=\sum_{i=1}^{N}\Delta g_{i}^{\circ } \end{array}$$

For simplicity duplex initiation free energies have been neglected here. Based on the duplex sequence we can now calculate the duplex binding constant

(7) $$K=\frac{Z_{D}}{Z_{P}Z_{T}}=\frac{Z_{D}}{e^{\Delta G_{D}^{\circ }/RT}}$$

### Consideration of Point Defects

We introduce a defect parameter $\Delta g_{def}^{\circ }$ (a simplified description of the mismatch NN parameters in [7,10]) to account for the point defect at the defect position *x *(a similar approach is described in [25]).

An analytical derivation of the DPI for homopolymer sequences shows that the partition function (provided as a function of defect position – see Eq. 8) is increased for defects located near the duplex ends.

(8) $$Z_{D}(x)=Z_{D_{PM}}+\left(e^{\frac{(N-x)\Delta g^{\circ }}{RT}}+e^{\frac{x\Delta g^{\circ }}{RT}}\right)\left(e^{\frac{\delta \Delta g_{def}^{\circ }}{RT}}-1\right)$$

In Eq. 8 the defect impact $\delta \Delta g_{def}^{\circ }=\Delta g_{def}^{\circ }-\Delta g^{\circ }$ has been factored out, revealing a general (defect-type independent) position dependence that is largely governed by the distance between the defect at position *x *from the duplex ends. Defects proximate to the duplex ends increase end-domain opening. The partition function is increased due to the number of thermally populated (partially denatured) duplex states. The defect destabilization $\delta \Delta g_{def}^{\circ }$ determines how far *Z*_*D *_is elevated in respect to the perfect match partition function (*Z*_*PM *_≈ 1) and thus how far the DPI propagates into the interior of the duplex. With Eqs. 7 and 8 we obtain an expression for the DPI on the duplex binding constant *K*(*x*).

(9) $$K=\frac{\left(e^{\frac{x\Delta g^{\circ }}{RT}}+e^{\frac{(N-x)\Delta g^{\circ }}{RT}}\right)\left(e^{\frac{\delta \Delta g_{def}^{\circ }}{RT}}-1\right)+1}{\left(e^{\frac{N\Delta g^{\circ }}{RT}}\right)\left(e^{\frac{\delta \Delta g_{def}^{\circ }}{RT}}\right)}$$

**Figure 5:**
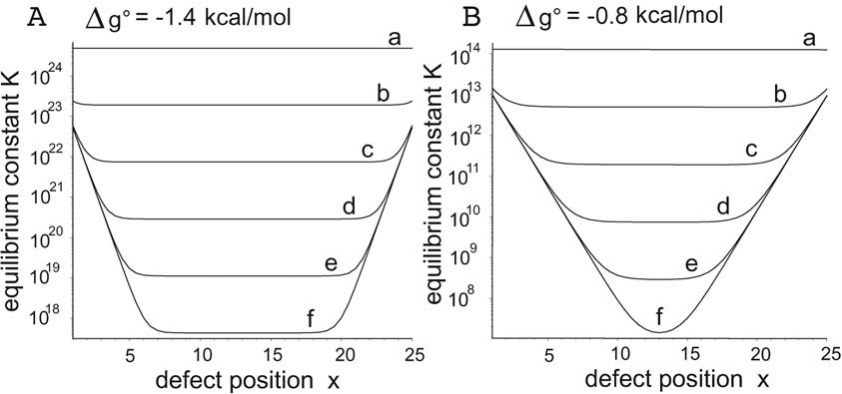
**Positional influence of single base MM defects on the duplex stability for two different NN pair free energies Δ*g*° at a temperature of 310 K**. Curves *a *to *f *correspond to defect destabilization values $\delta \Delta g_{def}^{\circ }$ of 0 to 5 kcal/mol (incrementally increased by 1 kcal/mol). Defect destabilization $\delta \Delta g_{def}^{\circ }$ is quoted per affected NN pair. (*A*) Δ*g*° = -1.4 kcal/mol, this corresponds to an average NN-pair free energy; (*B*) Δ*g*° = -0.8 kcal/mol corresponding to a weakly bound sequence of A·T and T·A base pairs.

Fig. 5 illustrates Eq. 9 for two different duplex stabilities.

While defects near the duplex ends result in low mismatch discrimination only (i.e. small reduction of *K *with respect to the PM binding affinity) defects in the center result in higher MM discrimination as *K *then approaches the value of the two-state equilibrium constant. NN-pair free energy increments $\delta \Delta g_{def_{37}}^{\circ }$ for single base MMs are in the range of 1 to 3 kcal/mol per NN-pair (derived from NN parameters [8,10]). Employing these values in Eq. 9 for Δ*g*° = -1.4 kcal/mol (Fig. 5A), DPI propagation is restricted to 3 or 6 NN-pairs, respectively. However, in subsequences with weakly bound NN-pairs (as demonstrated in Fig. 5B) the DPI can propagate further towards the middle of the duplex.

### Relation Between the Hybridization Signal and the Binding Free Energy Δ*G*_*D*_

In order to compare our numerical analysis to the experimentally observed hybridization signals we need to understand how the hybridization signal (fluorescence intensity from hybridized targets) is linked to duplex stability. As detailed below the assumption of a single (homogeneous) binding affinity within a microarray feature of the Langmuir adsorption model does not describe the experimentally observed hybridization signal intensities well. In this section we account for the heterogeneity that is introduced by *in situ *synthesis related random mutations of the microarray probe sequences.

The importance of the adsorption model for the description of microarray hybridization has been discussed previously in [36-39]. In the simplest description the equilibrium between single stranded probes and targets and hybridized duplexes *T *+ *P *⇄ *D *can be described by a Langmuir-type adsorption isotherm (Eq. 10). Under our experimental conditions targets were in sufficient excess, the target concentration [*T*] = [*T*_0_] can be taken as constant. Since the hybridization signal intensity is expected to be proportional to the fraction of hybridized probes *θ *= [*D*]/[*P*_0_] we will in the following refer to *θ *as the hybridization signal.

(10) $$\theta =\frac{\left[D\right]}{\left[P_{0}\right]}=\frac{K\cdot [T_{0}]}{1+K\cdot [T_{0}]}$$

Taking $K=e^{-\Delta G_{D}^{\circ }/RT}$ we obtain a sigmoidal relation between the hybridization signal and duplex free energy Δ*G*_*D*_.

(11) $$\theta =\frac{e^{-\Delta G_{D}^{\circ }/RT}\cdot [T_{0}]}{1+e^{-\Delta G_{D}^{\circ }/RT}\cdot [T_{0}]}$$

**Figure 6:**
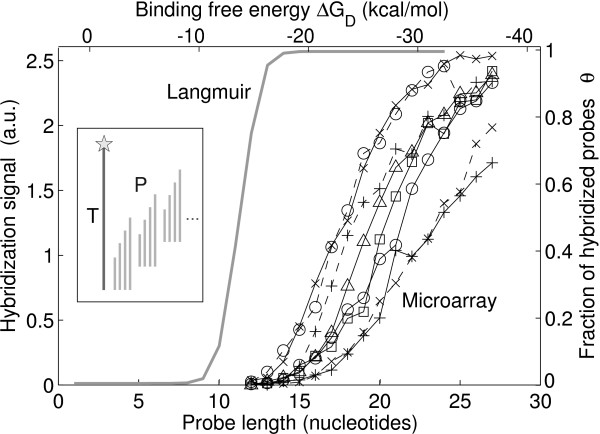
**Hybridization signal versus duplex stability**. The sigmoid transfer function *θ*($\Delta G_{D}^{\circ }$) of the Langmuir isotherm (*right scale*) has a narrow transition region ($\delta \Delta G_{D}^{\circ }$ ≈ 6 kcal/mol at a temperature of 310 K and a target concentration of 1 nM). Microarray hybridization signals (*left scale*) for incrementally increased duplex stabilities: The probe sequence motif was translated along the target sequence in increments of two bases (see *inset*), thus providing a set of different curves. All probes were hybridized with the common target sequence *URA *(1 nM in 5 × SSPE, for 20 minutes at 45°C). The approximately linear increase of the hybridization signal in the transition region extends over at least 13 base pairs ($\delta \Delta G_{D37}^{\circ }$ ≈ 20 kcal/mol).

Our experimental data suggest an approximately linear relation between the hybridization signal and the duplex binding free energies (within the free energy range covered by the defect profiles). However, with Eq. 11 an approximately linear relation between *θ *and Δ*G*_*D *_is only provided within a narrow range *δ*Δ*G*° ≈ 6 kcal/mol (at *T *= 310 K and [T_0_] = 1 nM). This cannot reproduce the experimentally observed DPI of the hybridization signal, since the free energy range of the defect profile exceeds the transition region. To investigate how the fluorescence intensity of hybridized targets is related to duplex stability on the microarray surface we performed a hybridization assay comprising sets of probes in which the probe length (assumed to be roughly proportional to duplex free energy) is incrementally increased (inset in Fig. 6). The experimental results in Fig. 6 show a sigmoid relation between the hybridization signal and probe length. However the transition region extends over at least 13 base pairs ($\delta \Delta G_{D37}^{\circ }$ ≈ 20 kcal/mol) over which a monotonous increase of the hybridization signal is observed. In agreement with our findings a linear relation between microarray hybridization signals (on spotted microarrays) and duplex binding free energies $\Delta G_{D}^{\circ }$ (derived from calorimetric measurements) has been reported recently by Fish *et al*. [40]. The large deviation from the Langmuir-equation agrees with previous observations [25,41]. An effective isotherm with a broadened transition region, a Sips-isotherm, has been reported [25,42,43] to provide a better description of surface hybridization on microarrays. This isotherm can result from a heterogeneous, gaussian distribution of binding affinities. Reasons given for the heterogeneity include variation of the probe local environment, surface electrostatics [44] and entropic blockage [45]. As we show in the following a major contribution to the heterogeneity of binding affinities is probe polydispersity [25,38,46,47], which is a result of sequence defects generated in the *in situ *synthesis process of DNA Chips, which introduces single base mismatches, base bulges and truncations.

Assuming a stepwise error rate of 10%, more than 90% of the 25 mer duplexes contain at least one synthesis error [13]. Since the number of synthesis errors per probe follows a binomial distribution, the majority of the strands contains between one and three single base defects.

We calculate binding constants *K*_*i *_of the individual, randomly "mutated" probe sequences on the basis of the zipper model. Using the approach of Forman *et al*. [48] we obtain the total hybridization signal by summing up over the distribution of probes, where the contribution of each individual mutated probe *θ*_*i *_is described by a Langmuir equation (Eq. 10) with the binding constant *K*_*i*_. Probe polydispersity (in length as well as in sequence) reproduces a "stretched isotherm" [47] (similar to a Sips isotherm), with a significantly broadened transition region. This explains our experimental results in Figs. 6 and 2 well. A simulation of the transfer function *θ*($\Delta G_{D}^{\circ }$) for various error rates and a comparison between the experimental data in Fig. 6 and the corresponding simulation results are provided in Additional file 1, Fig. S6.

### Numerical Analysis of Mismatched Duplex Stability-Comparison with Experimental Results

To model experimental results with the partition function approach we choose the NN free energy of the mismatched base pair $\Delta g_{def}^{\circ }$ as a free parameter. $\Delta g_{def}^{\circ }$ = 1 kcal/mol (at T = 325 K) describes our experimental observations (in particular the dominating positional influence with respect to defect type-related influences) best – see Fig. 3. This value is also in good agreement with bulk solution parameters [10]. Results of the numerical simulation (in Fig. 3A) demonstrate that the shallower slope of the hybridization signal at the right duplex-end corresponds to a series of weak NN pairs (as anticipated by Eq. 9). The partition function *Z*(*x*) largely determines the positional influence. Additionally, as shown in Fig. 3B, defect-type related influence (the difference between MM and PM free energies *δ*Δ*g*° affects the statistical weight of the completely dissociated duplex *w*_*D*_) is reflected in the hybridization affinity *K*(*x*) and in the hybridization signal *θ*(*x*). In addition to single base pair defects our binding affinity model reproduces well our experimental results on the binding affinities of oligonucleotide duplexes with two single base deletion defects (for details see Additional file 1, Fig. S5).

**Figure 7:**
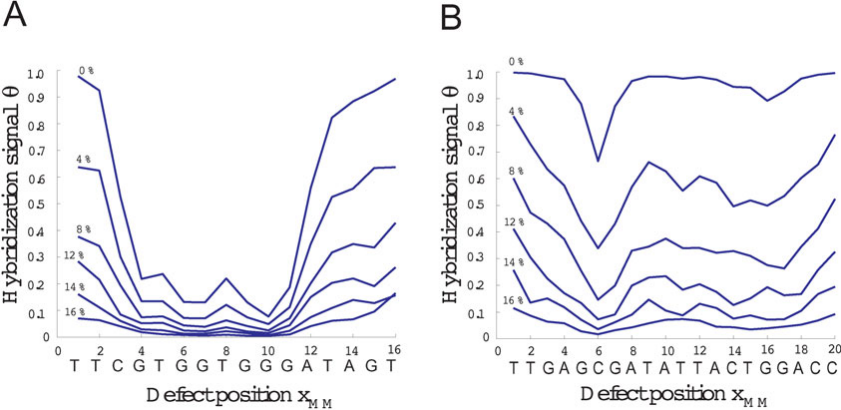
**Influence of the synthesis error rate on the shape of the single base mismatch defect profile**. The defect profiles (which correspond to the experimental data in Fig. 3 (*A*) and (*B*)) were calculated for error rates between 0 and 20 percent (per nucleotide coupling step). In (*A*) a positional influence is rather independent of the error rate – the duplex free energy range covered by the defect profile is within the approx. linear transition region. Whereas in (*B*) at an error rate of 0 percent, the free energy range of the defect profile doesn't match the transition region – the positional influence is hardly visible. At larger error rates the positional influence becomes dominating over the defect-type related influence.

In Fig. 7 we investigate the influence of heterogeneous probe-target binding affinities (see previous paragraph) on the shape of the defect profile. If the range of the mismatched duplex free energies is within the transition region we observe an approximately linear relation between the hybridization intensity and the binding free energy [40]. If the defect profiles free energy range exceeds the narrow transition region (like for example Fig. 7B, at an error rate of 0 percent) the positional influence remains hardly visible.

### Approximation of the Zipper Model with a Position Dependent Nearest-Neighbor (PDNN) Model

In order to investigate the generality of our finding, we investigate if PDNN models, which fit experimental data well, can be inferred from our model framework. We note that zippering has been previously proposed as the rationale behind the PDNN model in [25].

In the following we investigate the contribution of each base pair to duplex stability and ask if there is a position-dependent contribution of Watson-Crick NN pairs in the same way as for defects.

This idea is the basis of the PDNN model [17,21,41] in which $\Delta G_{D}^{\circ }$ is obtained as a position-dependent weighted sum of nearest-neighbor free energies.

(12) $$\Delta G_{D}^{\circ }=\sum_{i=1}^{N}w_{i}\Delta g_{i}^{\circ }$$

**Figure 8:**
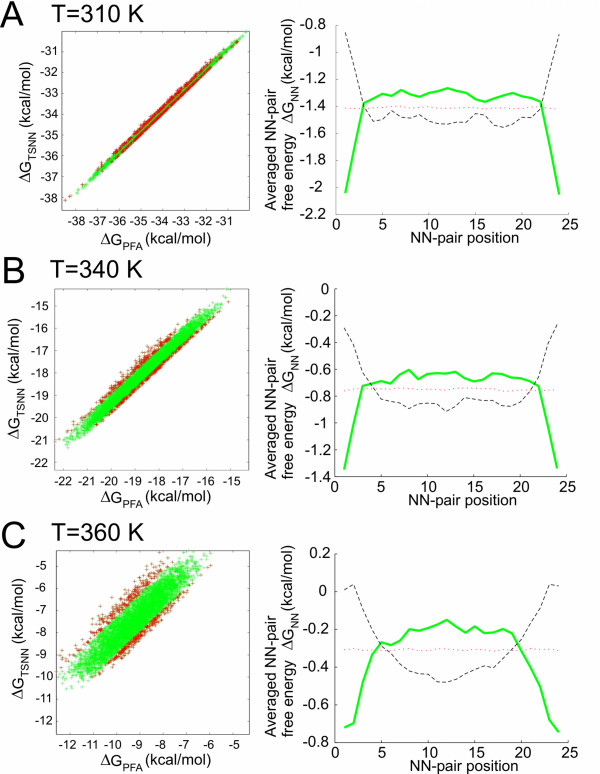
**Comparison of the two-state nearest neighbor (TSNN) model and the zipper model (partition function approach – PFA)**. To investigate for which sequences the difference between TSNN free energies and PFA free energies is largest, we have created a large set of 5000 random 25 mer sequences with a similar nucleobase composition. Scatter plots of TSNN free energies versus PFA free energies (*left*) show a very good correlation at a temperature of 310 K (*A*). At higher temperatures (340 K and 360 K shown in *B *and *C*) we find significant deviations between the two models. We have selected the 5% of sequences with the largest residuals (highlighted by *red symbols*) and determined the position-dependent distribution of NN free energies (shown *right*) by averaging (→ averaged NN pair free energy versus NN-pair position. The Gibbs free energies in upper, middle and lower plots refer to temperatures T = 310 K, 340 K and 360 K, respectively). At 310 K the sequences with the most stable Δ*G*_PFA _have their weak NN pairs at the outermost two base positions (*dashed black line*) and therefore the more strongly binding NN pairs in the interior. Vice versa sequences with the weakest Δ*G*_PFA _(*solid green line*) have strong NN pairs located at the outermost positions. The mean NN free energy (average over all sequences) is indicated by the *dotted red line*. At 340 K for the most stable sequences (according to PFA) the weakest NN-pairs are concentrated at the six outermost base positions (at each duplex end). At 360 K (which is above the melting temperature of the duplexes) the NN pair stabilities follow a parabolic position dependence.

Following our theoretical approach we create a set of 7500 oligonucleotide duplexes assembled from a given set of NN pairs. Although the TSNN (two-state nearest-neighbor) free energy of these duplexes is identical, the calculation with the zipper model indicates significant differences among the stabilities of the individual duplex sequences (see Additional file 1, Fig. S7). We investigate the positional distribution of NN pairs in the weakest/strongest 5% of the duplexes. We find that in the most stable duplexes the stronger NN-pairs are located in the center whereas in the least stable duplexes the strong NN-pairs are located near the duplex ends. This result has been reproduced with the partition function based UNAfold software (DINAMelt web server [9]) with excellent agreement to the zipper model. A similar investigation (see Fig. 8) employing a set of random duplexes composed of nonidentical NN-pairs confirms the result. In Additional file 1, Fig. S8 we show that duplex free energy values determined with the zipper model can indeed be approximated with a PDNN model. The positional weights – described by a parabolic function *w*_*i*_(*x*) – have their maximum in the middle of the duplex. The results in Figs. 8 and Additional file 1, Fig. S8 indicate that the contribution of the outer NN-positions to duplex stability decreases with increasing temperature. At 340 K the three outermost NN pairs (which is in total six of 24 NN pairs) have a significantly reduced contribution to duplex free energy. At a still lower temperature of about 310 K the positional weights converge to *w*_*i*_(*x*) = 1, which is equivalent to the TSNN model.

## Conclusion

In this paper we studied, experimentally and theoretically, the stability of short (*l *< 26 bp) linear surface-bound oligonucleotide duplexes with single base defects. We demonstrated that the rationale behind positional dependent models of oligonucleotide duplex stability is the partial denaturation of the duplexes. We have shown, that the strong influence of the defect position on mismatch discrimination [11-14,16,49] and the influence of the sequence context – beyond nearest neighbors [14,34] can be quantitatively inferred from a molecular zipper model. Partial (end-domain-)denaturation of the duplex as proposed by us in [26] as well as in [16,24,25] results in a positional influence that is entropic in nature. The zipping process is modulated by the sequential arrangement of the base pairs. The model confirms the observed influence of the sequence context beyond the nearest-neighbors. Further the zipper model provides a theoretical foundation to the positional dependent nearest-neighbor model of Zhang *et al*. [17].

In the commonly employed two-state nearest-neighbor model, nucleic acid duplex hybridzation/denaturation is considered to be an all-or-none process. According to literature indeed end-fraying effects are expected to be small beyond three bases [34], however, in our studied case, we conclude that end-fraying plays a non-negligible role. This is surprising since the dissociation probability of individual base pairs decreases towards the center of the duplex in an exponential fashion (see Additional file 1, Fig. S4) and remains very low for most NN-pairs.

We propose that the effect of the defect position on probe-target binding affinities becomes apparent in the hybridization signal intensities due to the unavoidable probe polydispersity of optical synthesis. It indeed appears that the positional dependence of single base MM discrimination is more commonly observed on photolithographically produced DNA oligonucleotide arrays [11-14] rather than (in large scale studies) on spotted microarrays [40,50,51] or in solution-phase experiments. We notice, however, that in small studies (investigating few sequences) a positional influence in solution [52] and on spotted microarrays [49] has been reported. The probe polydispersity in our experiments smoothes out the steep sigmoid relation between the hybridization intensity and binding free energy Δ*G*_*D *_that is expected for defect free probes, and explains why (within a relatively broad range of $\delta \Delta G_{D37}^{\circ }$ ≈ 20 kcal/mol) variations of the binding free energies – like for example the influence of the defect position – are reflected (by means of an approximately linear relation) in the hybridization signal intensities.

## Authors' contributions

TN developed the experimental setup and methods, carried out the experiments and statistical data analysis, computational modeling and drafted the manuscript. TM participated in the development of the experimental setup and in data interpretation and helped to draft the manuscript. JK and WM participated in the modeling and helped to draft the manuscript. AO conceived of the study, and participated in its design and coordination and aided in drafting the manuscript.

## Supplementary Material

Additional file 1 **Supplementary material**. We provide additional figures illustrating our experimental data and theoretical analysis. We further provide the detailed analytical derivation of the defect positional influence on homopolymer sequences. Click here for file
