# Supplementary material for: Position dependent mismatch discrimination on DNA microarrays – experiments and model
Source: BMC Bioinformatics. 2008 Dec 1;9:509. doi: 10.1186/1471-2105-9-509 (PMC2661940; doi:10.1186/1471-2105-9-509)
Supplement: Additional file 1 — Supplementary material. We provide additional figures illustrating our experimental data and theoretical analysis. We further provide the detailed analytical derivation of the defect positional influence on homopolymer sequences. [file 1471-2105-9-509-S1.pdf]

Supplementary Material

"Position dependent mismatch discrimination on DNA microarrays - experiments and model"

Thomas Naiser, Jona Kayser, Timo Mai, Wolfgang Michel, and Albrecht Ott

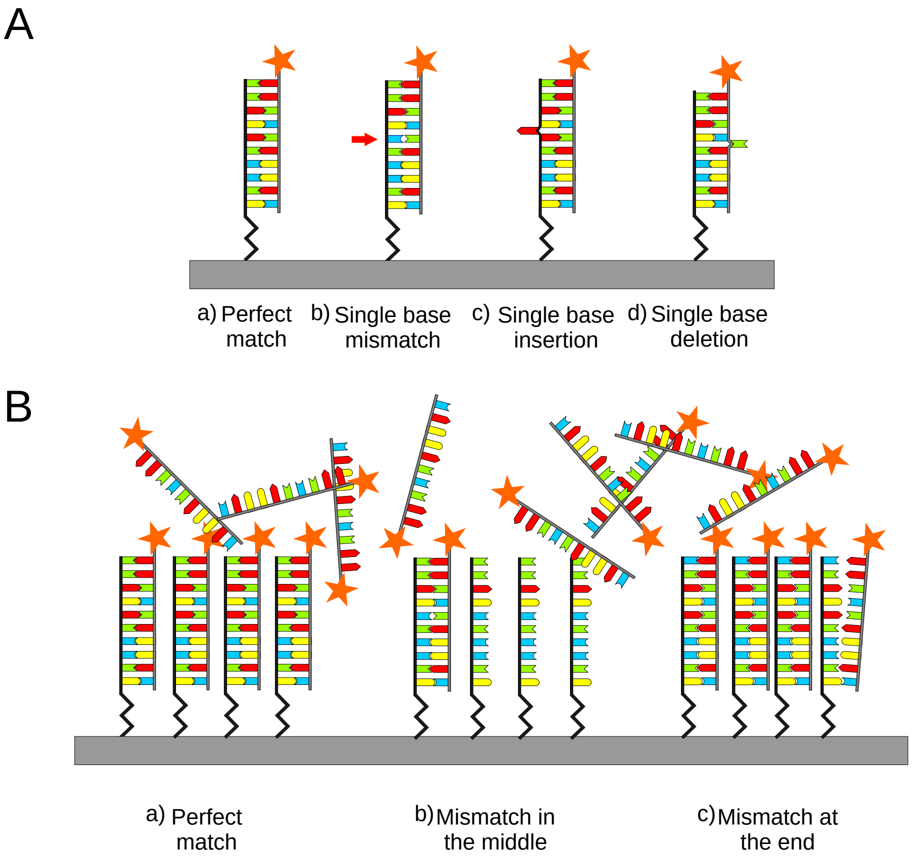

Figure S 1. Schematics of the microarray hybridization experiments. Each hybridization assay is performed with a single type of fluorescently labeled target oligonucleotide sequence. End-tethered Cy3-fluorophores are depicted by an orange star. Part *A* shows (*a*) a perfect matching (PM) duplex, (*b*) a single base mismatched (MM) duplex – resulting from a base substitution in the probe sequence (with respect to the PM probe sequence), (*c*) a bulged duplex resulting from a single base insertion in the probe sequence, and (*d*) a bulged duplex resulting from a single base deletion in the probe sequence. Part *B* depicts an ensemble of three microarray features: (*a*) with PM probes, (*b*) probes with a single base MM in the middle, and (*c*) probes with a single base MM at the duplex end. Differences in the binding affinities with respect to the target sequence are reflected by the density of hybridized target strands, and can thus be detected by means of fluorescence microscopy.

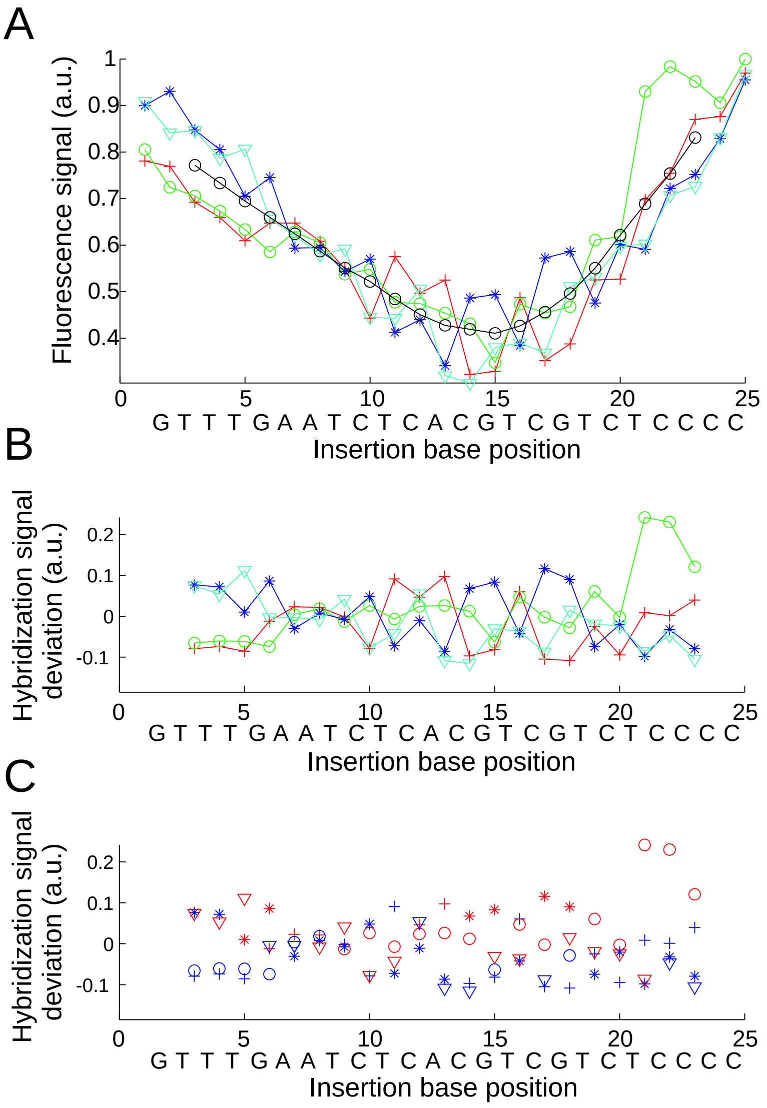

Figure S 2. Position dependent impact of various single base insertion defects on the hybridization affinity for the probe sequence motif 3'-GTTTGAATCTCACGTCGTCTCCCC-5' (hybridized with the complementary target sequence *URA*). (*A*) Hybridization signal versus base bulge position. Symbols: Insertion of A (*red crosses*), C (*green circles*), G (*blue stars*), T (*cyan triangles*). The moving average of all insertion types (*black line*) corresponds to defect positional influence (DPI). (*B*) The deviation of the hybridization signal from the moving average highlights defect type related influence. (*C*) Group II bulges - resulting from insertions next to like bases (→ degenerate bulges) - display significantly increased hybridizations signals (*red symbols*) compared to that of nondegenerate Group I bulges (*blue symbols*).

Analytical Derivation of the Defect Positional Influence on a Homopolymer Sequence

We investigate DPI on a homogeneous sequence with nearest neighbor free energies Δg°. The defect free energy is Δg°_{def}. The free energy difference between the defective duplex and the perfect matching duplex is denoted by $\delta\Delta g^\circ_{def} = \Delta g^\circ_{def} - \Delta g^\circ$. For MM defects $\delta\Delta g^\circ_{def}$ is distributed over two nearest neighbor pairs.
Defects only affect statistical weights of partially denatured states $S_{k,l}$ if the defect position x is located in the unzipped section of the duplex (Fig. S3 *A*). Thus the partition function Z of a single defect duplex can be separated in two parts (see also Fig. S3 *B*):

- Z_A(x) comprises states not affected by the defect
- Z_B(x) comprises states affected by the defect

We can now factor out the type dependent impact of the defect:

$$Z(x) = Z_A(x) + Z_B(x)\ e^{(\Delta g^\circ_{def} - \Delta g^\circ)/RT} \tag{1}$$

Equivalently, using the partition function of the perfect match duplex $Z_{PM} = Z_A(x) + Z_B(x)$ we can also write:

$$\begin{aligned} Z(x) &= Z_A(x) + Z_B(x) - Z_B(x) + Z_B(x)\ e^{(\Delta g^\circ_{def} - \Delta g^\circ)/RT} \\ Z(x) &= Z_{PM} + \Delta Z(x) \end{aligned} \tag{2}$$

Thus

$$Z(x) = Z_{PM} + Z_B(x)\ (e^{(\Delta g^\circ_{def} - \Delta g^\circ)/RT} - 1) \tag{3}$$

Summing up the statistical weights $w_{k,l} = e^{\Delta g^\circ (k+N-l)/RT}$ over all partially denatured states, we obtain:

$$\begin{aligned} Z(x) = & \\ & \sum_{k=0}^{N-1} \sum_{l=k+1}^{N} w_{k,l} + \left(\sum_{k=0}^{x-1} \sum_{l=k+1}^{x} w_{k,l} + \sum_{k=x}^{N-1} \sum_{l=k+1}^{N} w_{k,l} \right) \cdots \\ & \cdots \times \left(e^{\frac{\Delta g^\circ_{def} - \Delta g^\circ}{RT}} - 1 \right) \end{aligned} \tag{4}$$

This can be approximated by:

$$Z(x) \simeq Z_{PM} + \left(e^{\frac{(N-x)\Delta g^\circ}{RT}} + e^{\frac{x \Delta g^\circ}{RT}} \right) \left(e^{\frac{\delta \Delta g^\circ_{def}}{RT}} - 1 \right) \tag{5}$$

Probe and target strands each take half of the duplex dissociation energy. Since we assume that the single stranded species exist only in an unfolded state the partition functions for probes and targets are approximated by

$$Z_P = Z_T = e^{\Delta G_D^\circ/(2\cdot RT)} = e^{N\Delta g^\circ/(2\cdot RT)} e^{\delta\Delta g_{def}^\circ/(2\cdot RT)} \tag{6}$$

With $Z_{PM} \approx 1$ and $K = Z/(Z_P \cdot Z_T)$ we obtain the duplex binding constant

$$K = \frac{\left(e^{\frac{x\Delta g^\circ}{RT}} + e^{\frac{(N-x)\Delta g^\circ}{RT}}\right)\left(e^{\frac{\delta\Delta g_{def}^\circ}{RT}} - 1\right) + 1}{\left(e^{\frac{N\Delta g^\circ}{RT}}\right)\left(e^{\frac{\delta\Delta g_{def}^\circ}{RT}}\right)} \tag{7}$$

The influence of the sequence composition (also considering the position of NN pairs) on single base MM discrimination is demonstrated in Fig. S4: Here the asymmetrical composition of the sequence motif $(C\cdot G)_{12}(A\cdot T)_{12}$ results in an asymmetric positional dependence of the MM discrimination.

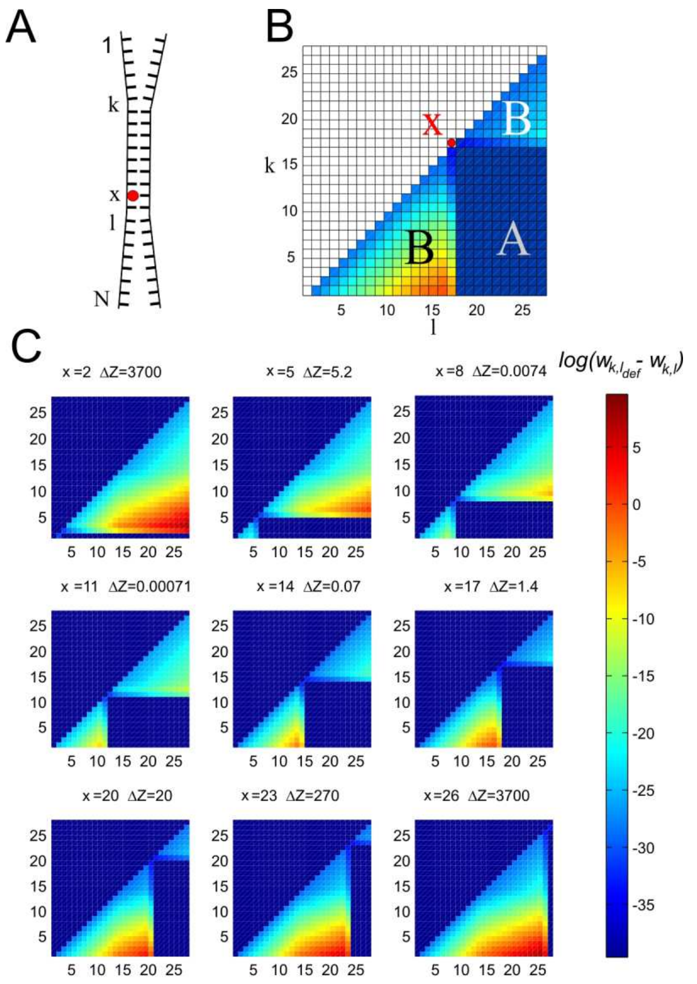

Figure S 3. Impact of a point defect at position x on the statistical weights of the microstates - a comparison with the statistical weights of the corresponding perfect matching (PM) duplex. (A) Depending on defect position x and on the positions k and l of the two zipper forks, a defect either affects the free energy (and thus the statistical weight $w_{k,l_{def}}$ of the partially denatured microstate $S_{k,l}$, or not. (B) The statistical weights of the partially denatured duplex states are arranged in matrix form. Matrix elements correspond to microstates $S_{k,l}$ of the duplex $(C{\cdot}G)_{12}(A{\cdot}T)_{12}$. For visualization the colors of the individual matrix elements show (the logarithm of) the difference between MM duplex statistical weights and the corresponding PM duplex statistical weights log $(w_{k,l_{def}} - w_{k,l_{PM}})$. In the area A, corresponding to microstates in which the defect is embedded in the hybridized duplex section microstates are unaffected, hence the difference of statistical weights is zero. In region B microstates are affected by the defect: Here, in respect to the perfect matching reference, free energies $\Delta G_{k,l}$ are modified by the amount $\Delta g_{def} - \Delta g_{PM}$. Destabilization by the defect results in increased statistical weights of partially unzipped microstates. Part (C) shows the matrix depicted in (B) for different defect positions x. Defects near the duplex ends (e.g. $x = 2$ or $x = 26$) result in significantly larger partition function values in comparison to defects in the center of the duplex (e.g. at x=14).

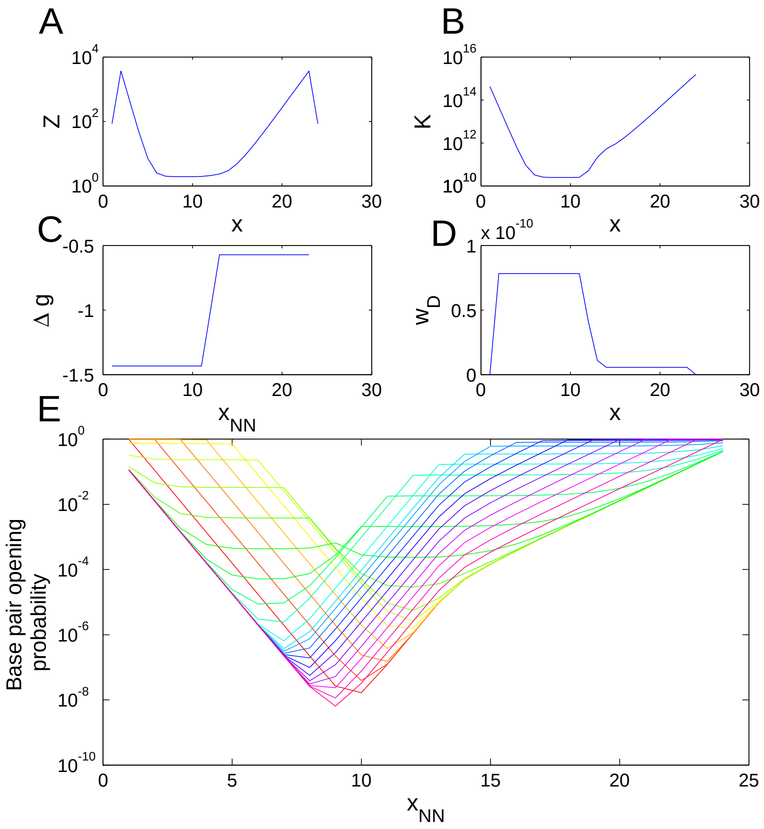

Figure S 4. Influence of single base MM position on the stability of the asymmetric duplex (C·G)$_{12}$(A·T)$_{12}$. Partition function approach with parameters T=330 K; $\Delta g^{\circ}_{def\,37} = 2$ kcal/mol. (*A*) Partition function of the duplex $Z(x)$ as a function of defect position x. The slope $dZ(x)/dx$ is steeper towards the left end of the duplex (consisting of strongly bound CC/GG nearest neighbor pairs) than towards the right side of the duplex, which is comprising the weaker AA/TT pairs. The origin of the position dependence of $Z(x)$ is depicted in Fig. S3.
(*B*) The logarithmic plot of the duplex binding constant $K(x)$ (Eq. 8) reflects the strong impact of defect position on the hybridization signal observed experimentally (a comparison with experimental data is shown in Fig. 5). Duplex stability is least for defects located in the center of the duplex. The position dependence of the binding constant originates largely from the intramolecular partition function $Z(x)$. (*C*) shows the arrangement of the NN pair stabilities Δg in the nucleic acid sequence. (*D*) The statistical weight of the completely dissociated state is equivalent to the inverse of the equilibrium constant in the two-state NN model. Variations of curve (*D*) reflect variations of $\delta\Delta g_{def}$ and are thus dependent on the defect type. In the profile of binding constants $K(x)$ (*B*) defect type dependent influences originating from the statistical weight $w_D(x)$ of the completely dissociated duplex are significantly smaller than the positional influence introduced by the duplex intramolecular partition function $Z(x)$. The plot of the base pair dissociation probabilities (*E*), as anticipated, shows an exponential decrease towards the center of the duplex. The exponent, which is determined by the sequence of nearest neighbor parameters, is significantly different for the two sections of the asymmetric sequence. Defect position, encoded by the color spectrum - ranging from *red* (defect at the left end) to *violet* (defect at the right end) - also recognizable by a sharp kink - strongly affects partial denaturation of the duplex. In respect to defects in the strongly bound section defects in the weakly bound section (in the left half of the duplex) enable the duplex to occupy a larger number of partially unzipped states. The increased partition function Z results in higher duplex stabilities.

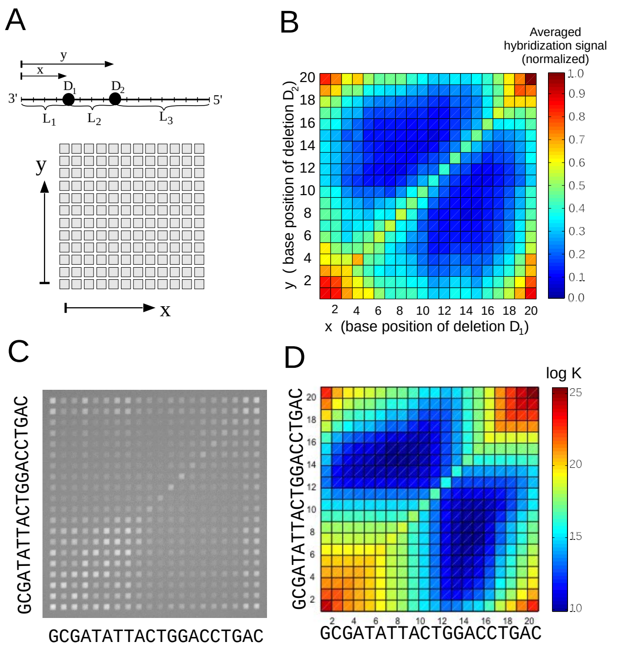

Figure S 5. Binding affinity of short linear duplexes containing two single base deletions at varying positions x and y. Comparison of experiment and model. (A) Microarray probe with two single base deletions D_1 and D_2 at positions x and y, respectively. The two defects divide the sequence into three subsequences of length L_i. The probe set comprising all configurations (x,y) of the two defects (in duplicate) is arranged as a compact array of microarray features. Feature positions (x,y) correspond to the defect positions x and y within the sequence. Experimental results in (B) show the averaged hybridization signal intensities of 20mer probes with two deletion defects (the diagonal corresponds to single base deletions). Since hybridization signals show defect type related variations (depending on the type of NN pairs affected) we have averaged hybridization signals from 9 different probe sequence motifs. The largest hybridization affinity is observed if defects are both located a the same end or at opposite ends. The destabilization is largest if defects are separated by 5-6 base positions. We found that the hybridization signal I depends strongly on the lengths L_i of the subsequences between the defects and can be fitted well (not shown) by the function $I \sim \sum L_i{}^{1.4}$. Defects at the surface-bound 3'-end (base position 1) affect the hybridization signal slightly less then defects at the 5'-end. This may, however, be related to bias originating from the chosen set of probe sequence motifs. Each microarray hybridization assay was performed with a single target oligonucleotide (1 nM) in 5×SSPE, 0.01% Tween-20 at a temperature of 30 to 40°C. (C) Fluorescence micrograph showing the hybridization signal intensities for the probe sequence motif 3'-GCGATATTACTGGACCTGAC-5'. (D) shows the corresponding binding affinities as calculated with the zipper model. The color scale is proportional to the logarithm of the binding constant K. We observe a good agreement with the experimentally obtained hybridization signal in (C).

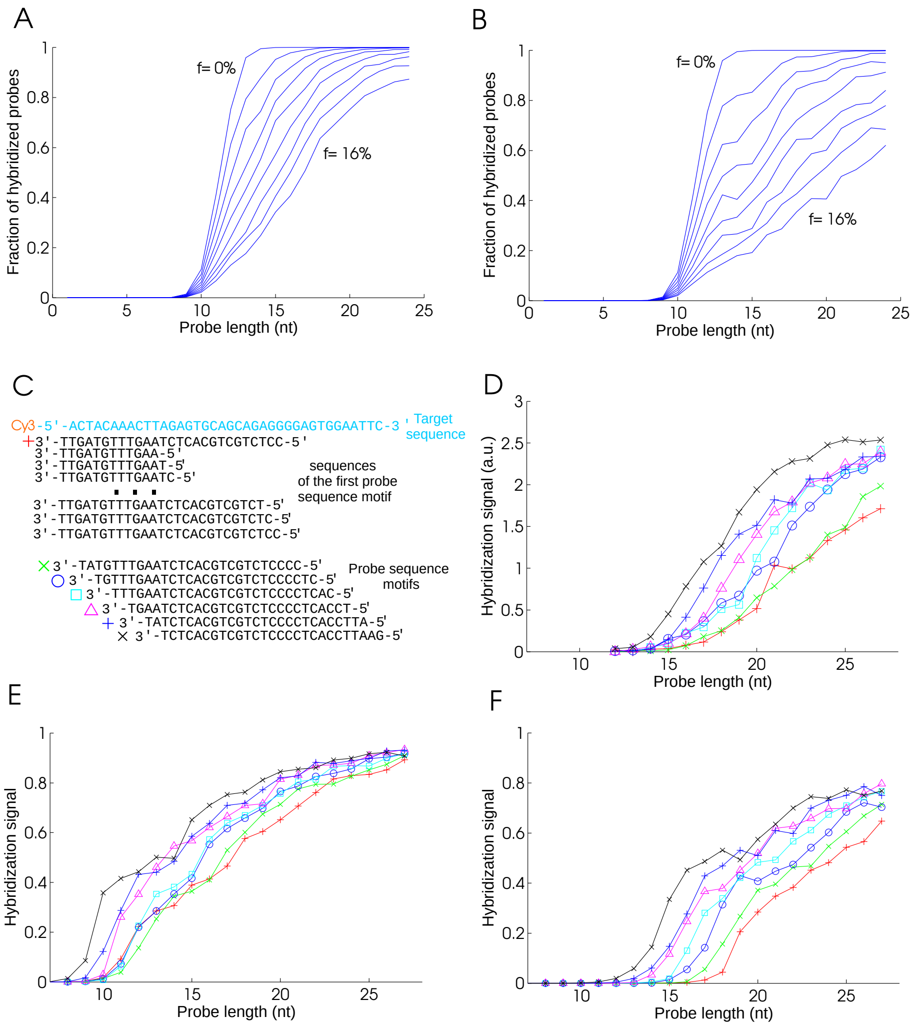

Figure S6. Numerical simulation of the influence synthesis errors on the hybridization signal. Assuming all duplexes within a microarray feature have a uniform binding affinity, the transfer function $\theta(\Delta G^{\circ}_{D})$ is described by a Langmuir isotherm. θ, the fraction of hybridized probes is tantamount to the hybridization signal, and ΔG°_{D} is the duplex binding free energy. Using the partition function approach we have calculated hybridization signals for distributions of probe sequences containing various synthesis defects - similar to those expected in a single microarray feature. In (A) and (B) the probe length l_P (assumed to be roughly proportional to ΔG°_{D}) is varied between 6 and 25 base pairs. The resulting relationship $\theta(l_P)$ basically describes the transfer function $\theta(\Delta G^{\circ}_{D})$. Synthesis error rates f (fraction of errors per synthesis step) have been varied between 0% and 16% in steps of 2%.

The simulation code assumes single base MM defects with a $\Delta g^{\circ}_{def} = -0.5\,\mathrm{kcal/mol}$ in (A), and $\Delta g^{\circ}_{def} = 2\,\mathrm{kcal/mol}$ in (B). As can be seen, the relatively weak defects in (A) like the strong defects in (B) result in a significant broadening of the transfer function in respect to the narrow transition range for f=0% (which is corresponding to the Langmuir isotherm). Parts (C) to (F) compare experimental results (shown in D - identical with Fig. 6) to the simulation results. (C) For a series of probe sequence motifs (translated in steps of two base positions along the complementary target sequence) we have generated several sets of probe sequences (sequence motifs shown) with incrementally increasing length. Experimentally obtained hybridization signals of the corresponding series are shown in (D). Part (E) shows the corresponding simulation results, taking into account synthesis errors (parameters: T=310 K, target concentration=1 nM, error rate f=10%, ΔG°_{D}=2 kcal/mol). Significantly improved agreement with the experimental results is achieved in (F) using an increased temperature T=333 K and a reduced error rate f=6%. Like in the experiment the minimum probe length required for hybridization is about 12 to 16 nt.

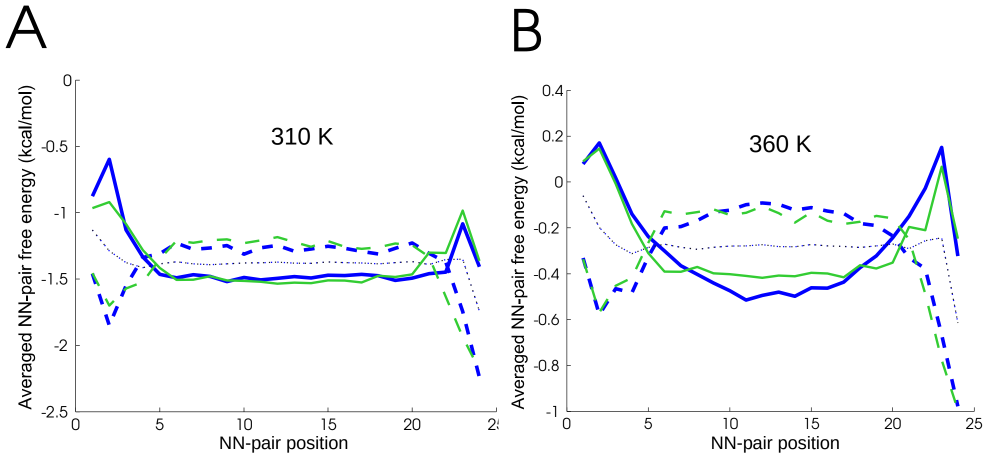

Figure S7. We performed a theoretical investigation on duplex stability with a set of 7500 randomly created 25mer sequences with an identical NN pair content. For those sequences, owing to identical NN pairs the two-state nearest-neighbor model predicts identical duplex binding free energies. Similar as in Fig. 7 we determined those sequences with the largest/smallest ΔG_{PFA} and determined for these subsets the spatial distribution of NN pairs (NN pair stability versus NN pair position). NN free energies are referring to 310 K (A) and 360 K (B), respectively. For the most stable 5% of the duplexes the average NN free energies are plotted as a function of NN pair position (*solid blue curve*), the NN pair distribution of the least stable 5% is shown by *dashed blue curve.* The *dotted line* shows the average nearest neighbor free energy distribution over all 7500 sequences. In parallel we employed the partition function based software UNAFold (8) to determine the melting temperatures of the duplexes. We selected the 5% of the sequences with the highest/lowest melting temperatures and established the corresponding NN pair distribution (highest melting temperatures: *solid green curve*; lowest melting temperatures: *dashed green curve*). We found a good agreement between the results of the PFA and UNAFold. At a temperature of 360 K the PFA shows a significantly stronger position dependence. The asymmetric bias at the duplex ends originates from the fact that the constraint of an identical NN pair content provides sequences with identical NN pairs at the duplex ends.

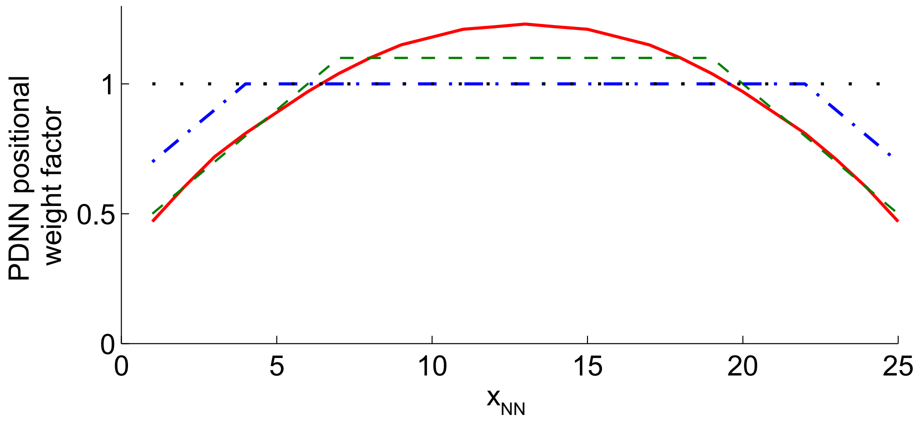

Figure S8. Approximation of the partition function approach (PFA) by a position dependent nearest-neighbor model (PDNN). For a set of one thousand 25mer random probe sequences (chosen for a similar nucleobase content) we compared perfect-match duplex free energies ΔG for the PFA, PDNN and two-state nearest-neighbor (TSNN) model. Using Pearson's correlation coefficient r_P for the correlation between ΔG_{PDNN} and ΔG_{PFA} and r_T for the correlation between the TSNN free energy ΔG_{TSNN} and ΔG_{PFA}, we found that the best fit PDNN weight function $w(x_{NN})$ strongly depends on temperature. At T=360 K a parabolic weight function (*solid red line*) reproduces the PFA results significantly better ($r_P = 0.979$) than the two-state nearest neighbor model ($r_T = 0.892$). Approximating the parabolic function by a composed function (*dashed green line*) of decreasing ramps towards the edges and constant weights in the center, we obtain the same Pearson coefficient. However, since the temperature of 360 K is chosen significantly above the melting temperature of the 25mer duplexes (which is approx. 340 K according to the two-state NN-model), this shouldn't be considered a physical result. At T=340 K the best fit ($r_P = 0.996$ versus $r_T = 0.981$) is achieved with a similar composed function with reduced weight parameters (ramps) only at the three outermost base positions (*blue dashed-dot line*). Towards lower temperatures the PFA converges towards the TSNN model. At 340 K the weight parameters for 360 K provide a relatively poor fit ($r_P = 0.946$). At 310 K the PFA results match that of the TSNN ($w(x_{NN}) = 1$) almost perfectly ($r_T = 0.999$). Thus, we have shown that with increasing temperatures – towards the melting transition – the distribution of NN pairs plays an increasingly important role for duplex stability.
